# CRYSTAF, DSC and SAXS Study of the Co-Crystallization, Phase Separation and Lamellar Packing of the Blends with Different Polyethylenes

**DOI:** 10.3390/polym15081940

**Published:** 2023-04-19

**Authors:** Xuerong Yao, Ying Shi, Yujing Tang, Chunxia Luo, Liping Hou, Minqiao Ren, Cui Zheng, Li-Zhi Liu

**Affiliations:** 1 SINOPEC (Beijing) Research Institute of Chemical Industry Co., Ltd., Beijing 100013, China; tangyj.bjhy@sinopec.com (Y.T.); luocx.bjhy@sinopec.com (C.L.); houlp.bjhy@sinopec.com (L.H.); renmq.bjhy@sinopec.com (M.R.); zhengc.bjhy@sinopec.com (C.Z.); 2 Advanced Manufacturing Institute of Polymer Industry, Shenyang University of Chemical Technology, Shenyang 110142, China; shiying@syuct.edu.cn (Y.S.); violetlj@yahoo.com (L.-Z.L.)

**Keywords:** PE blends, co-crystallization, fractionation, structure, CRYSTAF, SAXS

## Abstract

The crystallization of polyethylene (PE) blends is a highly complex process, owing to the significant differences in crystallizability of the various PE components and the varying PE sequence distributions resulting from short- or long-chain branching. In this study, we examined both the resins and their blends through crystallization analysis fractionation (CRYSTAF) to understand the PE sequence distribution and differential scanning calorimetry (DSC) to investigate the non-isothermal crystallization behavior of the bulk materials. Small-angle X-ray scattering (SAXS) was utilized to study the crystal packing structure. The results showed that the PE molecules in the blends crystallize at different rates during cooling, resulting in a complicated crystallization behavior characterized by nucleation, co-crystallization, and fractionation. We compared these behaviors to those of reference immiscible blends and found that the extent of the differences is related to the disparity in crystallizability between components. Furthermore, the lamellar packing of the blends is closely associated with their crystallization behaviors, and the crystalline structure varies significantly depending on the components’ compositions. Specifically, the lamellar packing of the HDPE/LLDPE and HDPE/LDPE blends is similar to that of the HDPE component owing to its strong crystallizability, while the lamellar packing of the LLDPE/LDPE blend is approximately an average of the two neat components.

## 1. Introduction

Polyethylene (PE) is the most popular plastic in the world and one of the most widely used polymers because of its superior properties and relatively low cost. Different types of PE with different molecular architectures have been commercialized over the past 80 years from different polymerization processes and catalysts [1]. The three main types of PE are high-density polyethylene (HDPE), linear low-density polyethylene (LLDPE), and low-density polyethylene (LDPE). HDPE is almost linear with no branching or small amounts of short-chain branching to improve the environmental stress crack resistance. LLDPE resins made by incorporating higher levels of alpha-olefin comonomer have higher levels of short-chain branching. The LDPE resins have both long-chain branching and short-chain branching resulting from the free radical polymerization process [1,2].

In practice, many commercial PE resins are mixtures of polyethylene with varying branch types and distributions [2,3,4,5]. To achieve the desired properties, dual reactors are often used in the polyethylene industry to obtain a blend of different polyethylenes [1]. Additionally, liquid–liquid phase separation has been reported for certain LLDPEs [6,7,8,9] because of their broad branch content distribution. Even for LLDPEs with the same comonomer content, the branch distribution can vary significantly. For example, the sequence distribution of LLDPE made with a metallocene catalyst is typically more uniform than those made with a Ziegler–Natta catalyst [10]. Characterization techniques such as crystallization analysis fractionation (CRYSTAF) [4,11,12,13,14] and temperature rising elution fractionation (TREF) [4,15,16,17] have been developed to demonstrate the varying composition distributions of polyethylene and other semi-crystalline polymers [18,19], based on the relative crystallizability of their molecules.

On the other hand, for industrial applications, over 70% of PE is used as a blend composition to maximize its overall properties [20,21]. Examples of such blends include HDPE/LDPE blends [22,23,24], HDPE/LLDPE blends [23,24,25,26], and LLDPE/LDPE blends [27,28,29]. Therefore, studying the miscibility, crystallization, and morphology of PE blends is crucial for PE industrial applications [30]. Various experimental methods have been utilized to study PE blends, including small-angle neutron scattering (SANS) [31,32,33], small-angle light scattering (SALS) [34,35,36], rheology investigations [37,38,39], differential scanning calorimetry (DSC) studies [40,41,42,43,44,45,46,47], small-angle X-ray scattering (SAXS) [33,35,48], transmission electron microscopy (TEM) [49,50,51], crystallization analysis fractionation (CRYSTAF) [52,53], temperature rising elution fractionation (TREF) [52,54], and others. With the development of investigation methods and diversification of polyethylene blend species, scientists have deepened their understanding of polyethylene blends [30,31,32,33,34,35,36,37,38,39,40,41,42,43,44,45,46,47,48,49,50,51,52,53,54,55]. Combining various methods can enable a more comprehensive research of PE blends [33,34,56,57].

In PE blends, the interaction between components during crystallization is crucial not just for determining the final crystalline structure, but also for the strength of inter-crystalline connections. This is important for resistance to low temperature impacts, creep, slow crack growth, tear, and puncture [58,59,60,61,62,63]. The crystallization process and resulting crystalline structures of PE blends heavily depend on the difference in crystallizability of the components and their miscibility [30,31,32,33,34,35,36,37,38,39,40,41,42,43,44,45,46,47,48,49,50,51,52,53,54,55,56,57]. Co-crystallization between components of PE blends during the crystallization process was discussed early on by RG Alamo [40,41], MJ Hill [42], and others. It is generally accepted that co-crystallization occurs more easily when the structure of the components is more similar and happens more readily under a fast cooling rate. Previous publications have only touched on the nucleation effect in PE blends, including LDPE/HDPE [64,65], HDPE/very low-density polyethylene [51], and LLDPE/LDPE blends [66,67]. A more comprehensive discussion of the crystallization kinetics of PE blends is needed.

In the present work, different bulk PE materials containing HDPE, LLDPE, and LDPE were first studied by differential scanning calorimetry (DSC), crystallization analysis fractionation (CRYSTAF), and small-angle X-ray scattering (SAXS). The goal was to understand the non-isothermal crystallization behavior, the sequences distribution based on crystalline ability, and the crystal packing structure for each material. Selected types of PE blends, including HDPE/LDPE, HDPE/LLDPE, and LLDPE/LDPE blends, were then further investigated to better understand the crystallization behaviors and how one component affects the crystallization of another. CRYSTAF and DSC were used to study these effects, and SAXS was employed to analyze the lamellar structures. This research attempted to draw a complete picture of the crystallization behaviors and lamellar structures of the PE blends of different types, while also making inferences about the miscibility between components.

## 2. Materials and Methods

### 2.1. Preparation of PE Blends with Different Types of PE

Different types of PE consisting of HDPE, LLDPE and LDPE were used for blending. Information on the resins is listed in Table 1. The mass-average molecular weight ($\bar{M}$_w_) and the molecular weight dispersity (***Đ***_M_ *=* $\bar{M}$_w_ /$\bar{M}$_n_, $\bar{M}$_n_ is the number-average molecular weight) [68] of the resins were obtained by gel permeation chromatography (Model PL-GPC 220, Agilent Technologies, Santa Clara, CA, USA) using 1,2,4-trichlorobenzene as a solvent at 150 °C. A narrow distribution polystyrene standard sample was used for universal calibration. The branching content was determined by solution ^13^C nuclear magnetic resonance experiments performed on an AVANCEIII-400 MHz spectrometer (Bruker, Billerica, MA, USA) with a 10 mm probe at 125 °C. All sample solutions were prepared with 200 mg of the polymer material dissolved in 2.5 mL of d_4_-o-dichrolobenzene in a 10 mm tube at 130 °C. Occasional stirring was necessary to achieve a homogeneous solution. Two HDPE and two LLDPE resins with different compositions were used in the present work, as listed in Table 1. “HDPE-1” is a resin for blow molding, while “HDPE-2” is for pipe applications with a relatively high ***Đ***_M_. The “LLDPE-1” resin was made with a metallocene catalyst while the “LLDPE-2” resin was produced by a Ziegler–Natta catalyst. The density data at 23 °C were obtained from the producers together with the products.

PE blends were prepared in a Haake twin-screw extruder (Thermo Fisher Scientific, Waltham, MA, USA) with a melting temperature of 240 °C, including the HDPE-2/LDPE 50/50 blend, the HDPE-2/LLDPE-2 50/50 blend, and the LLDPE-2/LDPE 50/50 blend in a ratio of weight percent. The screw diameter was 16 mm and the screw ratio was 40:1. The screw speed was set as 150 r/min. Different types of neat PE also underwent the same twin-screw melt extruding process for comparison. The PE blends and neat PE after processing were hot-pressed into sheets of 1 mm thickness for subsequent differential scanning calorimetry (DSC) experiments. The samples were molded at 160 °C and subsequently quenched to room temperature within 30 s.

### 2.2. Differential Scanning Calorimetry (DSC)

The crystallization behaviors of the neat PEs and the blends in bulk were studied by DSC (Model Q100, TA Instruments, New Castle, DE, USA). The DSC instrument calibrated with indium was employed under a high purity nitrogen atmosphere and samples weighed about 10 mg. Each sample was first heated from 25 °C to 180 °C at 10 °C/min and held at 180 °C for 5 min to eliminate the thermal history, and then cooled at 10 °C/min to 25 °C, held at 25 °C for 0.5 min, and again heated to 180 °C at 10 °C/min. Cooling and second heating curves of the neat PEs and the blends were recorded. In addition, as reference blends, hypothesized totally immiscible blends of the neat components were introduced for comparison, the DSC curves of which were calculated by taking the average DSC profiles of the neat polymers [47]. The heat of fusion ($\Delta H_{m}$) of the samples was obtained from the second scan of the DSC thermogram. The degree of crystallinity X was calculated by the formula $X=\frac{\Delta H_{m}}{\Delta H_{0}}\cdot 100\%$, where $\Delta H_{0}$ is the enthalpy of melting of the completely crystallized PE (293 J/g [69]).

### 2.3. Crystallization Analysis Fractionation (CRYSTAF)

Crystallization kinetics of the neat PEs and three binary PE blends in dilute solution were studied by crystallization analysis fractionation (CRYSTAF) [11,12,13,14]. A CRYSTAF model 200 (Polymer Char, Valencia, Spain) was used for fractionation. The studied sample (30 mg) was dissolved in 1,2,4-trichlorobenzene (40 mL) at 160 °C for 60 min and then kept at 95 °C for 45 min to ensure complete dissolution. To collect data during the solution crystallization, the solution was gradually cooled to 35 °C at a rate of 0.1 °C/min. The crystallization at each temperature was measured by monitoring the PE concentration in the solution with an online IR5 detector. Additionally, reference blends consisting of completely immiscible combinations of the neat components were introduced for comparison. CRYSTAF curves for these blends were calculated by averaging the CRYSTAF profiles of the neat polymers.

### 2.4. Small-Angle X-ray Scattering (SAXS)

The crystalline structures of the neat PEs and the blends were investigated by SAXS [70,71]. Small pieces of about 10 mg were cut from the 1 mm sheets of the neat PEs and the three binary PE blends. These pieces were treated with the DSC instruments having the same thermal profile as the DSC non-isothermal crystallization study. Specifically, the pieces were heated to 180 °C and kept at 180 °C for 5 min, and then cooled to 25 °C at a cooling rate of 10 °C/min. After treatment with the DSC instruments, the pieces were used for crystalline structure study by small-angle X-ray scattering (SAXS) at room temperature. The SAXS measurements were carried out using a NANOSTAR U SAXS instrument (Bruker, Billerica, MA, USA) equipped with a heating unit. The X-ray source was an IμS-type generator operated at 40 kV and 650 μA. The wavelength was 0.1542 nm (CuKα). The scattering intensity was detected by a two-dimensional detector with 1024 × 1024 pixels and 100 μm pixel size. The distance between the detector and the sample was 1059 mm. Some X-ray experiments were conducted using synchrotron radiation with λ = 0.154 nm at Beamline 1W2A of the Beijing Synchrotron Radiation Facility (Beijing, China). Mar165-CCD was used for data collection. All data were corrected for air background before any analysis.

## 3. Results and Discussion

### 3.1. Crystallization-Driven Phase Separation of PE Molecules Due to Large Difference in Branching Degree

**Figure 1:**
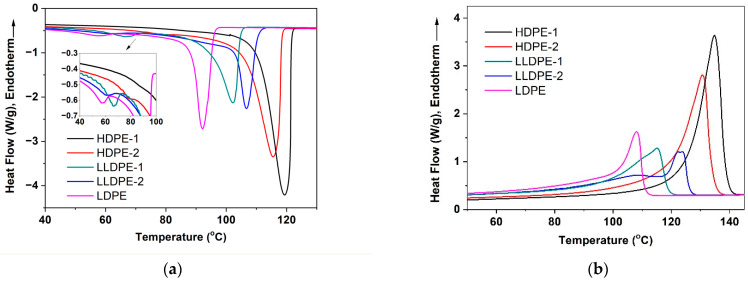
DSC cooling (10 °C/min) curves (**a**) and subsequent heating curves (**b**) of different PEs studied in this work.

The non-isothermal crystallization of the five different PEs was studied first by the DSC technique. As shown in Figure 1a, during a cooling process at a rate of 10 °C/min, the crystallization occurred at very different temperatures for the different types of PE. As seen from Figure 1a and listed in Table 2, all the PE resins showed a main crystallization peak at a high temperature and a small crystallization peak at a much lower temperature below 90 °C, although the small peak of the HDPE-1 was very weak.

Among the PE resins, HDPE-1 with a lower branching content of 0.4 per 1000C (as shown in Table 1) exhibited the highest crystallization temperature during the same cooling process (Figure 1a and Table 2). In contrast, HDPE-2 with 7.5 times higher branching content than HDPE-1 (Table 1) showed a broader main crystallization peak at a lower temperature (115.5 °C) than HDPE-1 because of the impact of the significantly higher branching degree, which slowed down the nucleation and crystallization of the HDPE. The two LLDPE resins, made with hexene and octene comonomer, respectively, had an approximately 4 times larger branching degree than HDPE-2 (Table 1), resulting in much lower major crystallization temperatures (102.4 °C and 107.2 °C for LLDPE-1 and LLDPE-2, respectively). LDPE molecules typically have both short-chain and long-chain branches owing to the radical polymerization reactions (Table 1). As a result, LDPE resins exhibited much weaker crystalline ability than LLDPE and crystallized at a much lower temperature (93.3 °C) during the same cooling process (Figure 1a).

The weak crystallization peaks observed in all the PE resins could be the result of a small fraction of the highly branched PE molecules unable to participate fully in the main crystallization process at high temperatures. Instead, these molecules were relegated to the interlamellar or inter-spherulite regions where they crystallized at a much lower temperature [3,65]. That is, the crystallization of the PE resins led to the phase separation of the small fraction of highly branched PE molecules from the major PE content and crystallized separately at a significantly lower temperature. In general, the temperature of the small peak of polyethylene resin with high branching content was relatively low. The temperatures of the small crystallization peaks in the two HDPE resins were higher than those in the LLDPE and LDPE resins. Moreover, the small crystallization peak in the HDPE-1 resin was quite weak because of the low branching content and limited amounts of highly branched PE molecules in the resin. For the two LLDPEs, the location of the small crystallization peak was also related to the catalyst and the comonomer distribution. Although the total branching content of LLDPE-2 was slightly lower than that of LLDPE-1, PE molecules with higher branching content were expected to exist in the LLDPE-2 resin owing to the much broader branch distribution of LLDPE-2 made by the Ziegler–Natta catalyst than the LLDPE-1 prepared by the metallocene catalyst. As a result, the temperature of the small crystallization peak in LLDPE-2 (61.7 °C) was lower than LLDPE-1 (66.4 °C) while the temperature of the main crystallization peak in LLDPE-2 (107.2 °C) was higher than LLDPE-1 (102.4 °C).

Figure 1b represents the melting thermograms obtained from subsequent heating (10 °C/min) of the neat PE materials studied in this work. It can be seen from this figure that the melting behaviors of the PE materials were very different. The HDPE samples displayed a significantly narrower melting peak with the highest melting temperature (135.5 °C for HDPE-1 and 130.7 °C for HDPE-2) compared to the other samples, indicating the thicker and more uniform lamellar stacks formed in these HDPE samples. The LLDPE demonstrated a broader melting peak with the presence of a melting shoulder than other samples, because of a broad distribution of short-chain branching. The temperatures at which extreme values occurred in the melting curve are listed in Table 2 as the melting point (*T*_m_). The melting curve of LLDPE-2 made with the Ziegler–Natta catalyst was even broader than that of LLDPE-1 made with the metallocene catalyst. Three extreme temperatures appeared in the melting curve of LLDPE-2, as listed in Table 2. The melting point of the LDPE resin was the lowest (107.5 °C). The calculated crystallinity is listed in Table 2 for all the materials. The two HDPE resins showed much higher crystallinity than the LLDPE resins and the LDPE resin owing to much lower branching content (Table 1).

### 3.2. Correlation of PE Sequence Distribution from CRYSTAF and Lamellar Packing from SAXS for HDPE, LLDPE, and LDPE

**Figure 2:**
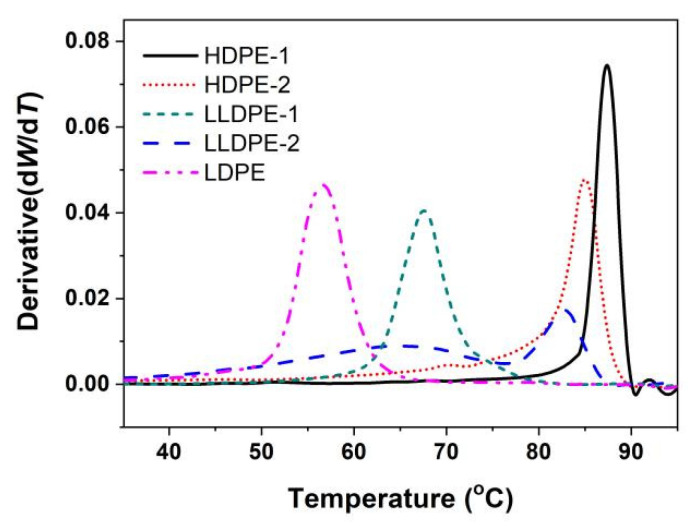
CRYSTAF profiles of the neat HDPE, LLDPE, and LDPE.

It is well known that CRYSTAF (crystallization analysis fraction) and TREF (temperature rising elution fractionation) are separation techniques based on the crystalline ability of polyethylene. Both techniques can fractionate PE resins based on different crystalline abilities by cooling the polymer solution slowly, though temperature rising elution fractionation requires a subsequent heating process (elution step) to obtain the information on polymer composition [4]. In this work, the distribution of the PE sequence was first analyzed by CRYSTAF for each resin used. As shown in Figure 2, both the neat HDPE resins had the strongest crystallizability and crystallized at a relatively high temperature range under identical experiment conditions. The resin HDPE-1 crystallized even more strongly because of a narrower composition distribution and less branching than HDPE-2 (Table 1). Compared with the HDPE resins, the two LLDPE resins crystallized at a lower temperature owing to much higher branching content (Figure 2). In fact, the crystalline ability of LLDPE could also be very different, as shown in Figure 2, where the LLDPE-1 resin made with the metallocene catalyst showed a narrow crystalline peak while the LLDPE-2 exhibited a very broad crystalline peak. In fact, the LLDPE-2 had a bi-model composition that showed a crystalline peak at the same high temperature as the HDPE-2 and a broad crystalline peak at a low temperature, covering the crystallization temperature range of both the LDPE and LLDPE-1, as shown in Figure 2. The neat LDPE studied in this work had the weakest crystalline ability and crystallized at the lowest temperature because of long-chain and short-chain branching.

It is seen in Figure 1 and Figure 2 that a stronger crystalline ability represented by the peak at the higher temperature from CRYSTAF corresponded to a higher melting point for the bulk crystallization observed by DSC. This difference was evident between HDPE-1 and LDPE. A broader distribution in crystalline ability seen from CRYSTAF led to a broader melting peak for the bulk crystallization from DSC, such as LLDPE-2 in Figure 1 and Figure 2. However, the crystalline ability distribution from CRYSTAF did not always have a good correlation with DSC melting for the bulk crystallization, such as a well-defined peak was observed from CRYSTAF for both LLDPE-1 and the LDPE, but a broad melting tail at low temperature range was observed for both materials. In the present work, the correlation between crystalline ability analysis from CRYSTAF and lamellar packing from bulk crystallization was also investigated.

**Figure 3:**
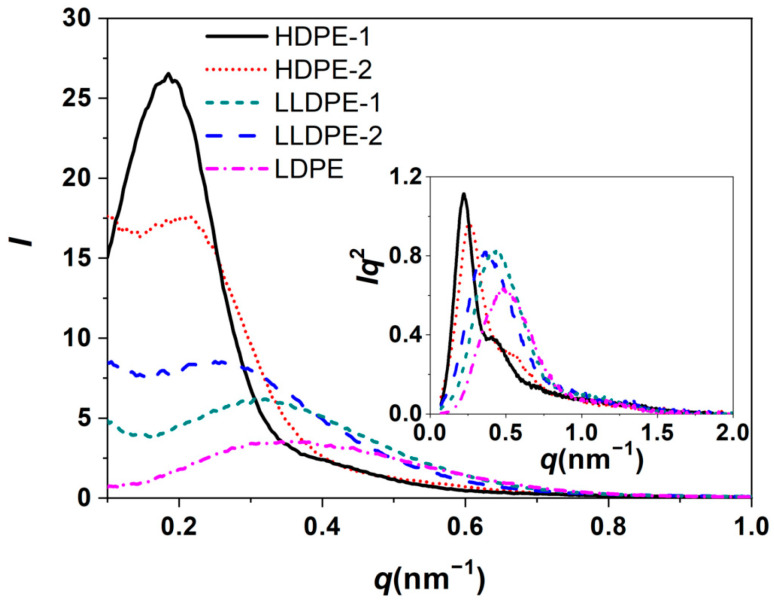
Linear and Lorentz-corrected SAXS profiles of the neat PEs used in this work.

The lamella structures of various types of PE were studied by small-angle X-ray scattering (SAXS). The linear and Lorentz-corrected SAXS profiles of different PEs are shown in Figure 3. The scattering peaks of the two HDPE resins were much narrower, and the scattering peaks of the two LLDPE resins and the LDPE resin were much broader. The broader SAXS peaks of the LLDPE resins were due to broader composition distributions, while the broader SAXS peak of the LDPE resin was due to the long-chain and short-chain branching of the PE molecules. The peak position ($q_{max}$) is related to the long spacing (*L*) corresponding to the average distance between neighboring crystalline lamellae via the Bragg equation [70,71].

(1) $$L=\frac{2\pi }{q_{\max}},$$

As seen in Figure 3, the $q_{\max}$ value of the two HDPE resins was the smallest, while that of LDPE was the largest. It was suggested that the two HDPE resins had the largest long spacing, while LDPE had the smallest long spacing. The Lorentz-corrected SAXS profiles of the two HDPE resins exhibited a discernible shoulder peak, which may be attributed to the second-order scattering of the lamellae [43]. At the same time, it was found that the SAXS peak of the HDPE-1 was more well-defined and narrower than the HDPE-2.

**Figure 4:**
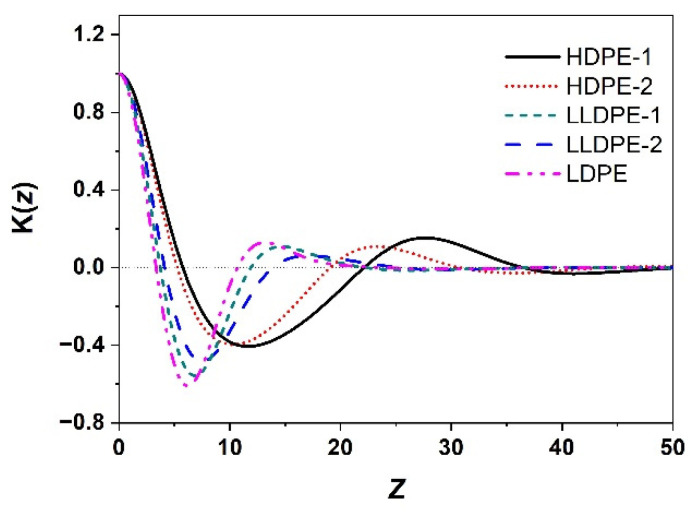
One-dimension correlation functions of the neat PEs used in this work.

The respective average thickness of the crystalline and non-crystalline regions can be calculated from the one-dimension correlation function K (*z*) as follows [46,57,70].

(2) $$K\left(z\right)=\left(1/K\left(0\right)\right)\int_{0}^{\infty }q^{2}I\left(q\right)\cos\left(qz\right)dq,$$

where *z* is the correlation distance along the direction from which the electron density distribution is measured. The average long spacing (*L*), the average crystalline phase thickness (*L*_c_) and the average non-crystalline phase thickness (*L*_a_) can be calculated from the correction function. The correlation functions of different types of PE are shown in Figure 4, and the obtained structural parameters are listed in Table 3. The *L*_c_ of the HDPE resins (20.1 nm for HDPE-1 and 16.5 nm for HDPE-2) were much higher than that for the neat LLDPE (5.3 nm for LLDPE-1 and 5.7 nm for LLDPE-2) and LDPE (4.9 nm), as the linear PE molecules in HDPE formed a thicker lamella than the branched ones in LLDPE and LDPE, resulting in a significantly higher melting temperature.

In fact, Figure 1 and Figure 4 exhibit a strong correlation between the CRYSTAF profiles of different polyethylenes, which represent the distribution of their macro-molecular crystalline ability, and their corresponding correlation function profiles that indicate the lamellar packing order in these samples. As shown in Figure 1, HDPE-1 exhibited a narrower distribution of crystalline ability than HDPE-2; a better lamellar packing structure (a better defined peak for correlation function), a larger long period, and thicker crystalline lamellae were observed for HDPE-1 in Figure 4. Similarly, the linear PE sequence in LLDPE-1 had a much narrower distribution than LLDPE-2 based on the CRYSTAF study (Figure 1); thus, a better correlation peak of lamellar packing was observed in LLDPE-1 than LLDPE-2 (Figure 4). LDPE had a narrower distribution in the linear PE sequence compared to the two LLDPEs, as observed from crystalline ability data obtained by CRYSTAF (Figure 1). Hence, the LDPE also exhibited a more ordered lamellar packing structure with a better defined correlation peak compared to the LLDPEs (Figure 4). That is, a narrower distribution of linear PE sequence was observed from CRYSTAF, thus a more ordered lamellar packing can be formed in the PE.

### 3.3. Crystallization of Different PE Component in a Mixture of Different Types of PE and Its Lamellar Packing

**Scheme 1:**
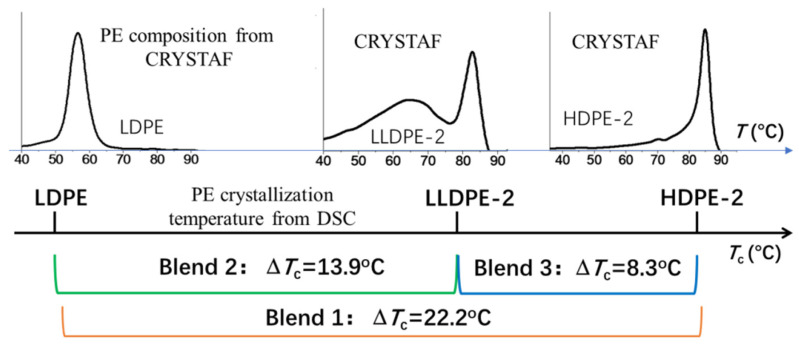
Illustration of the difference in crystallization temperature between the two PE components in the three different PE blends.

According to a report [4], co-crystallization between different types of PE is almost non-existent during CRYSTAF analysis of dilute PE solutions. However, the bulk crystallization of polyethylene is different; co-crystallization can occur among different types of polyethene [40,41,42]. In the present work, three different binary blends made with HDPE-2, LLDPE-2, and LDPE were studied. The DSC data in Table 2 indicated that the difference in the crystalline ability between HDPE-2 and the LDPE (Δ*T*_c_ = 22.2 °C) in the HDPE-2/LDPE blend was much larger than the two components in the LLDPE-2/LDPE blend (Δ*T*_c_ = 13.9 °C) and in the HDPE-2/LLDPE-2 blend (Δ*T*_c_ = 8.3 °C), as shown in Scheme 1. The three blends were studied by CRYSTAF, DSC, and SAXS techniques to investigate how the bulk crystallization and lamellar packing were associated with the overall PE sequence distribution observed by CRYSTAF.

In the following discussion of the CRYSTAF and DSC results of all three PE blends, hypothesized totally immiscible reference blends of the neat components were introduced for comparison. The curves of the reference blends were calculated by taking the average of the profiles of the neat components, as the components in these reference blends were assumed to have no interaction at all.

#### 3.3.1. Crystallization of PE with Different Composition and Crystal Packing Structure in the HDPE-2/LDPE 50/50 Blend

**Figure 5:**
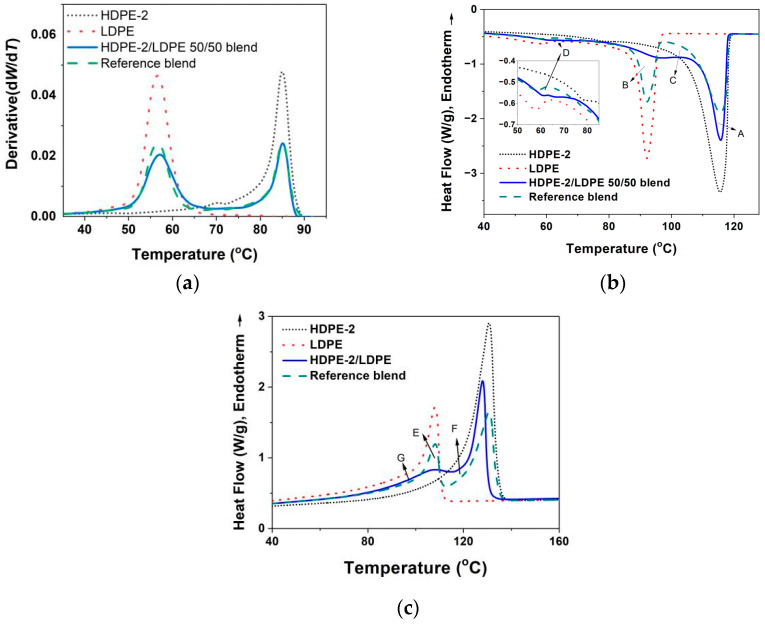
CRYSTAF profiles of the neat HDPE-2, the neat LDPE, and the HDPE-2/LDPE 50/50 blend (**a**), DSC cooling curve (**b**) and second heating curve (**c**) of the HDPE-2/LDPE 50/50 blend compared with neat component curves (dotted line) and calculated reference blend curves (dashed line). “A”–“G” in the figures indicates the area between the HDPE-2/LDPE 50/50 blend curve (solid line) and the reference blend curve (dashed line) at the corresponding locations.

The HDPE-2/LDPE 50/50 blend was first studied by CRYSTAF, as illustrated in Figure 5a, alongside CRYSTAF profiles of HDPE-2 and LDPE components. It is evident from Figure 5a that the CRYSTAF curve of the 50/50 HDPE-2/LDPE blend is almost identical to the calculated reference blend curve, suggesting that crystallization of the crystallizable PE sequence in such a dilute solution occurred independently based on its crystalline ability, and no co-crystallization could occur.

However, both the DSC cooling and heating thermograms for the bulk crystallization of the HDPE-2/LDPE blend were quite different from the calculated reference curve based on the individual components (Figure 5b,c). Compared with the calculated reference blend curve, the crystallization temperature of HDPE-2 in real HDPE-2/LDPE 50/50 blends was almost the same as the reference blend and neat HDPE-2, indicating that the presence of LDPE in the blend did not affect the crystallization kinetics of HDPE-2. However, the heat of the crystallization peak of HDPE-2 was noticeably greater than that of the reference blend (depicted by region “A” in Figure 5b). It is suggested that more PE molecules—probably LDPE with low branching content—crystallized at this temperature. These LDPE molecules can possibly co-crystallize with HDPE-2 or just crystallize at a higher temperature with the nucleation effect of HDPE-2. Additionally, it was also found that the melting temperature of HDPE-2 in the blend was 2.6 °C lower than that of neat HDPE-2 and the reference blend (Figure 5c and Table 4). This finding suggested that co-crystallization occurred between the HDPE-2 and LDPE components in the actual HDPE-2/LDPE 50/50 blend. Otherwise, the melting temperature of HDPE-2 in the blend would not change if the LDPE only crystallized with the nucleation effect of HDPE-2 without co-crystallization.

At the same time, a significant portion of LDPE in the actual blend was observed to crystallize at higher temperatures (region “B” to “C” in Figure 5b) than in the reference blend. It indicated that the crystallization of HDPE-2 had a nucleation effect on the crystallization of the LDPE with medium branching content. Accordingly, in the melting curve of the blend (Figure 5c), a significant portion of the LDPE crystals melted at a higher temperature (region “E” to “F”). It can be easily understood that the LDPE crystals formed at a higher temperature were more perfect, resulting a higher melting temperature.

In the low temperature range of the DSC cooling curve of the HDPE-2/LDPE blend, which was from 55 °C to 75 °C (region “D” in Figure 5b), there was a non-ignorable increase in the real blend compared with the reference blend. It can be easily understood that, apart from those portions of LDPE with low and medium branching content crystallizing at a higher temperature with the co-crystallization and nucleation effect of HDPE-2, LDPE molecules with high branching content were left and could only crystallize at a low temperature. There was also a slight increase in the low melting temperature range (region “G” in Figure 5c) in the DSC melting curve of the real blend compared with the reference blend because of the melting of the crystals formed at a low temperature.

**Figure 6:**
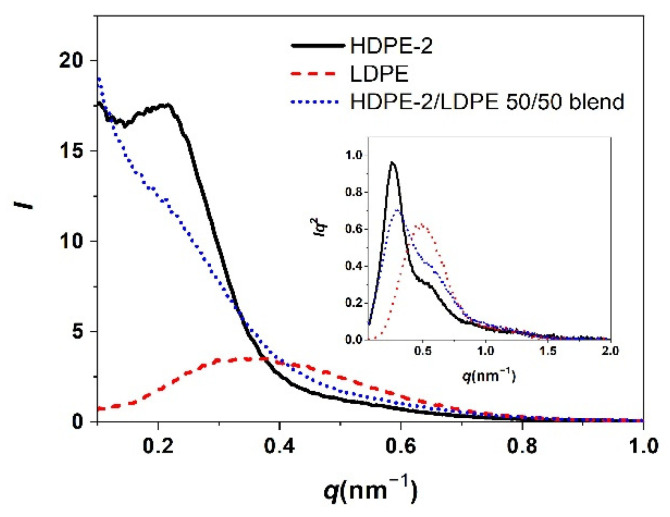
The linear and Lorentz-corrected SAXS profiles of the neat HDPE-2, neat LDPE, and the HDPE-2/LDPE blend.

**Figure 7:**
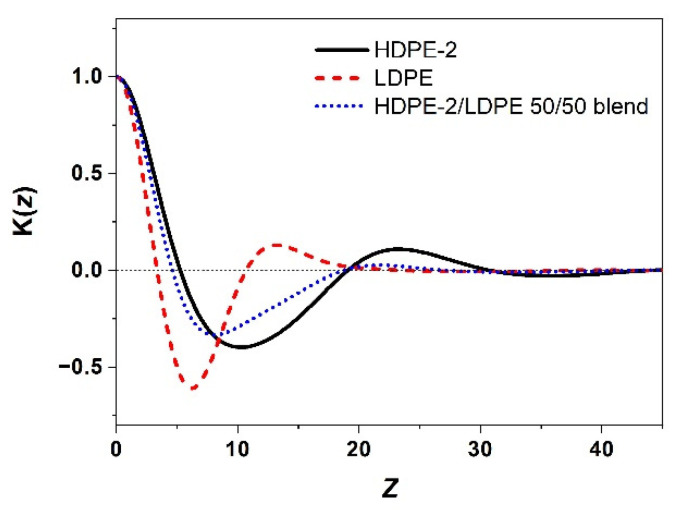
One-dimension correlation functions of the neat HDPE-2, neat LDPE, and the HDPE-2/LDPE 50/50 blend.

Based on the discussion above, the crystallization process of LDPE in its blend with HDPE-2 was fractionated by the behavior of its components and spread over different temperatures. Those LDPE molecules with low branching content could co-crystallize with HDPE-2 at the crystallization temperature of HDPE-2, while the LDPE molecules with medium branching content could crystallize at a higher temperature than neat LDPE with the nucleation effect of HDPE-2, and those LDPE molecules with high branching content were excluded and could only crystallize at a low temperature. In other words, the co-crystallization and the nucleation effect of HDPE-2 in the HDPE-2/LDPE 50/50 blend had a fractionation effect on the crystallization of LDPE. The existence of co-crystallization, the nucleation effect, and the fractionation effect of HDPE-2 on the crystallization of LDPE in the blend indirectly proved some extent of miscibility of the blend, as in the reference totally immiscible blend components will not influence each other. The crystalline structure of the HDPE-2/LDPE blend was further studied by SAXS and compared with the two neat components. The linear and Lorentz-corrected SAXS profiles are shown in Figure 6. The linear and the Lorentz-corrected SAXS profiles of the blend were similar to that of HDPE-2, rather than the average of the two neat components. This suggested that the HDPE-2 dominated the crystallization process in the blend. However, the peak of the blend in the linear or Lorentz-corrected SAXS profiles was obviously less well-defined than the neat HDPE-2. The one-dimension function of the blend is shown in Figure 7, and the average long spacing, the average thicknesses of the crystalline and non-crystalline phase were calculated and are listed in Table 5. The peak in the one-dimension function of the blend appeared much worse than in the neat HDPE-2 because of the broader composition distribution in the blend than the neat HDPE-2. The difference between the crystalline abilities of the neat HDPE-2 and the neat LDPE was large, as the crystallization temperature of the neat LDPE was 93.3 °C, which was 22.2 °C lower than that of the neat HDPE-2 (115.5 °C) (Table 2). Because of its branching, the neat LDPE formed very thin crystal lamellae (4.9 nm), while HDPE-2 formed much thicker lamellae (16.5 nm) (Table 5). Therefore, the lamellar stacks formed by the co-crystallization of HDPE-2 with LDPE would not be as regular or well-organized in terms of uniformity of the crystalline lamellar thickness. That was likely the reason for the absence of a well-defined SAXS peak for the HDPE-2/LDPE blend. The average *L*_c_ of the blend (15.9 nm) was slightly smaller than that of the neat HDPE-2 (16.5 nm), which was consistent with the lower melting temperature of the blend.

#### 3.3.2. Crystallization of PE with Different Composition and Crystal Packing Structure in the LLDPE-2/LDPE 50/50 Blend

**Figure 8:**
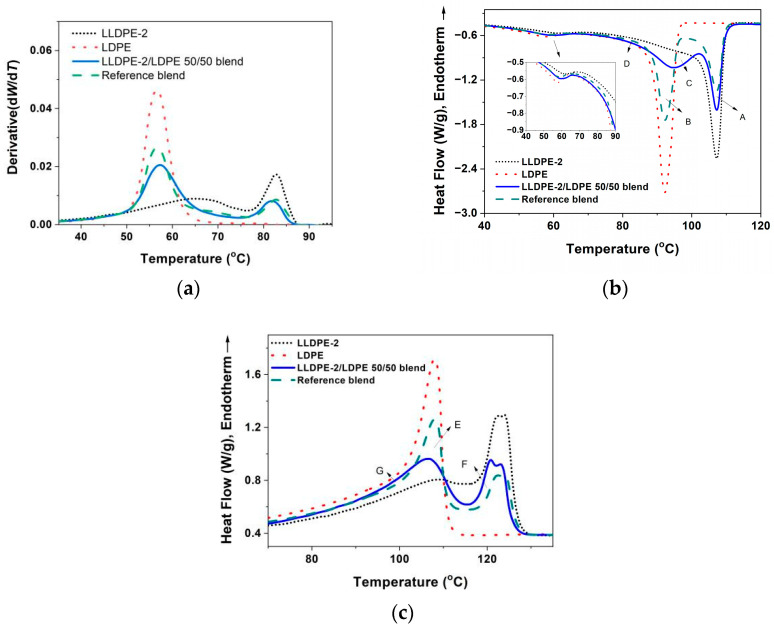
CRYSTAF profiles of LLDPE-2, LDPE, and LLDPE-2/LDPE 50/50 blend (**a**), DSC cooling curves (**b**) and subsequent heating curves (**c**) of the LLDPE-2/LDPE 50/50 blend compared with calculated reference blend curves. “A”–“G” in the figures indicates the area between the LLDPE-2/LDPE 50/50 blend curve (solid line) and the reference blend curve (dashed line) at the corresponding locations.

LLDPE/LDPE blends are very important for industrial applications since most polyethylene films are made of LLDPE/LDPE blends. In this work, the interaction between components in the crystallization of the LLDPE-2/LDPE blend was also first investigated by CRYSTAF (Figure 8a). The CRYSTAF curve of the 50/50 LLDPE-2/LDPE blend was almost identical to the calculated reference blend curve, again suggesting that crystallization in the dilute solution for each molecule was almost isolated.

However, both the DSC cooling and heating thermograms for the bulk crystallization of the LLDPE-2/LDPE blend were quite different from the calculated reference blend curves based the individual components (Figure 8b,c). The DSC data are listed in Table 6. The crystallization temperature of LLDPE-2 in the blend was almost the same as neat LLDPE-2 and the reference blend, indicating that LDPE did not significantly affect the crystallization kinetics of LLDPE-2. This finding was similar to that of the HDPE-2/LDPE blend. As the difference in *T*_c_ between the components was large enough, the component with weaker crystallizability could not significantly influence the crystallization kinetics of the component with higher crystallizability. The area of the crystallization peak of LLDPE-2 (region “A” in Figure 8b) was larger than that of the reference blend, which was similar to what occurred in the HDPE-2/LDPE 50/50 blend above. This result suggested that more PE molecules, probably low branching LDPE, crystallized at this temperature because of the nucleation effect of LLDPE-2 or co-crystallization with LLDPE-2. In the melting curves (Figure 8c), the melting temperature of LLDPE-2 in the blend was 0.8 °C lower than that in neat LLDPE-2 and the reference blend, indicating that co-crystallization between LLDPE-2 and LDPE components still occurred to some extent.

Compared with the calculated reference blend curve, a remarkable portion of the LDPE in the actual blend crystallized at a higher temperature together with the LLDPE-2 component (from region “B” to “C” in Figure 8b). This can be attributed to the nucleation effect of LLDPE-2 on LDPE with medium branching content, similar to the effect of HDPE-2 on LDPE in the HDPE-2/LDPE blends discussed earlier. Correspondingly, in the melting curves (Figure 8c), a significant part of LDPE melted at a higher temperature than the reference blend (from region “E” to “F”).

In the cooling curve of the blend, there was also slightly enhanced crystallization at a low temperature range of 60~88 °C (region “D” in Figure 8b). It is easy to understand that LDPE with low and medium branching content crystallized at higher temperatures with the nucleation effect of LLDPE-2 crystals or co-crystallization, while LDPE with high branching content was not crystallized at a low temperature. There was also an increase in the area of the melting curves at a low temperature range of 82~103 °C (region “G” in Figure 8c) compared with the reference blend, referring to the melting of the imperfect crystals forming at low temperature.

**Figure 9:**
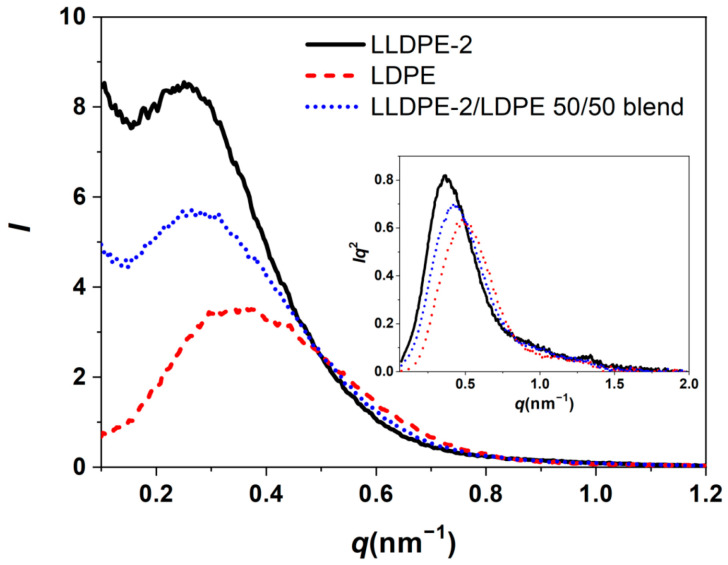
The linear and the Lorentz-corrected profiles of the neat LLDPE-2, the neat LDPE, and the LLDPE-2/LDPE blend.

**Figure 10:**
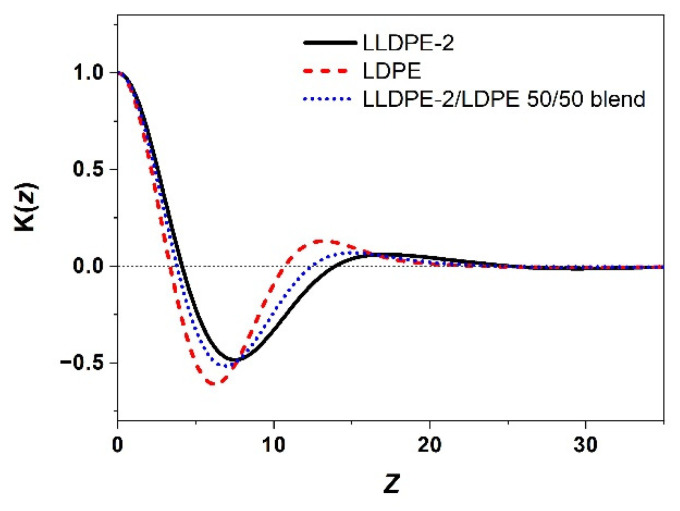
The one-dimension correlation functions of the neat LLDPE-2, the neat LDPE, and the LLDPE-2/LDPE blend.

In a word, the LDPE component in the LLDPE-2/LDPE blend showed fractionation-based crystallization with the co-crystallization and nucleation effect of LLDPE-2. LDPE molecules with low branching content can co-crystallize with LLDPE-2 at the crystallization temperature of LLDPE-2. LDPE with medium branching content can crystallize at a higher temperature than neat LDPE with the nucleation of LLDPE-2, and the LDPE molecules with high branching content can crystallize at a lower temperature. The influence between the components in the LLDPE-2/LDPE 50/50 blend again demonstrated the miscibility in the blend. The interaction between the components in the LLDPE-2/LDPE blend was similar to that of HDPE-2/LDPE, which consisted of co-crystallization, nucleation, and fractionation. The crystalline structure of the LLDPE-2/LDPE 50/50 blend was studied by SAXS. It was found in both the linear and Lorentz-corrected scattering intensity curves in Figure 9 that the LLDPE-2/LDPE blend showed a good scattering peak, indicating that well-packed lamellar stacks formed, which were also confirmed from the correlation function analysis in Figure 10 by the presence of a reasonably defined peak corresponding to the long spacing. Interestingly, the linear and the Lorentz-corrected SAXS profiles of the blend were approximately the same as the profiles of the neat LLDPE-2 and the neat LDPE samples (Figure 9). In addition, the peak of the linear or the Lorentz-corrected SAXS profiles was well-defined for the blend. The extent of co-crystallization between the LLDPE-2 and LDPE component was not expected to be large enough to form a regular co-lamella. If the lamellae of LLDPE-2 and LDPE were to mix together thoroughly, the peak of the SAXS profiles would not be well-defined. Therefore, it is likely that several periods of LLDPE-2 lamella and several periods of LDPE lamella components coexisted in the LLDPE-2/LDPE blend. The long spacing (*L*) and the average thickness of the crystalline phase (*L*_c_) were calculated by the one-dimension correlation functions in Figure 10 and are listed in Table 7. The *L* and the *L*_c_ of the LLDPE-2/LDPE blend were exactly the average of those of the two neat components.

#### 3.3.3. Crystallization of PE with Different Composition and Crystal Packing Structure in the HDPE-2/LLDPE-2 50/50 Blend

In this section of the study, we focused on the HDPE-2/LLDPE-2 50/50 blend. The difference in the crystallizability between components of the HDPE-2/LLDPE-2 blend (8.3 °C) was much smaller than the HDPE-2/LDPE blend (22.2 °C). The crystallization behaviors and crystalline structures and the interaction between components were expected to be different in the HDPE-2/LLDPE-2 blend compared with the HDPE-2/LDPE blend.

**Figure 11:**
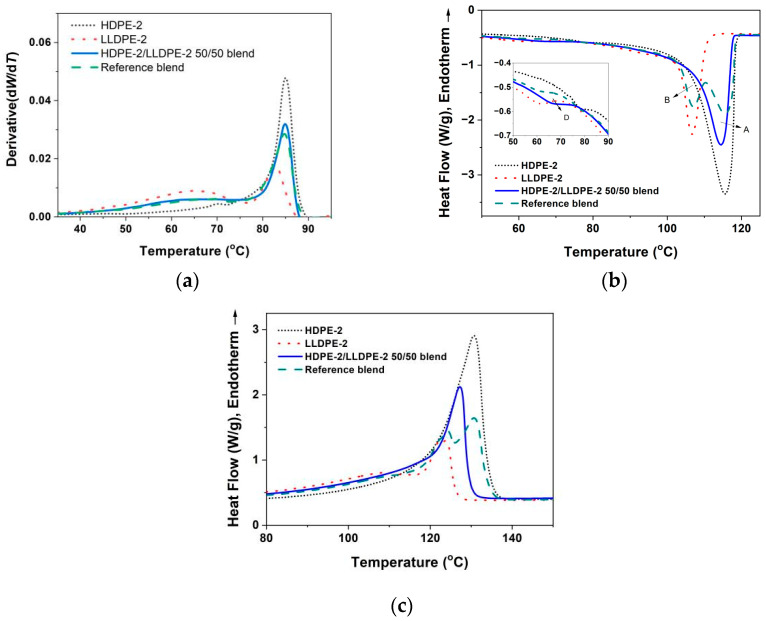
CRYSTAF curves of HDPE-2, LLDPE-2, and the HDPE-2/LLDPE-2 50/50 blend. (**a**) DSC cooling curves (**b**) and second heating curves (**c**) of the HDPE-2/LLDPE-2 50/50 blends compared with calculated reference blend curves. “A”/“B”/“D” in the figures indicates the area between the HDPE-2/LLDPE-2 50/50 blend curve (solid line) and the reference blend curve (dashed line) at the corresponding locations.

The CRYSTAF curves of the neat HDPE-2, LLDPE-2, and the HDPE-2/LLDPE-2 50/50 blends are given in Figure 11a. The CRYSTAF curve of the 50/50 HDPE-2/LLDPE-2 blend was almost coincident with the calculated reference blend curve, again suggesting that crystallization of the components in the solution was almost isolated.

However, both the DSC cooling and heating thermograms for the bulk crystallization of the HDPE-2/LLDPE-2 blend were quite different from the calculated reference blend curves (Figure 11b,c). As there were differences between both the crystallization temperatures and the melting temperatures of the two components (dotted lines), the calculated cooling and heating curves of the reference blend had two peaks, as shown with dashed lines in Figure 11b. However, the cooling curve and the melting curve of the real HDPE-2/LLDPE-2 50/50 blend showed only one main peak. It is noticed from Figure 11b,c and Table 8 that the blend’s crystallization temperature was 1.4 °C lower than that of the neat HDPE-2, and its melting point was 3.5 °C lower than that of neat HDPE-2. Additionally, the area of the main melting peak in the blend was notably larger than the HDPE-2 part in the reference blend. The lowered melting temperature and significantly higher crystallinity of HDPE-2 in the blend indicated co-crystallization of HDPE-2 and LLDPE-2 components because of the relatively smaller difference in crystalline ability of the LLDPE-2 with the HDPE-2. The melting temperature of the blend was almost the average of the melting temperature of neat HDPE-2 and neat LLDPE-2, indicating a large extent of co-crystallization between the HDPE-2 and LLDPE-2 components. As compared to the calculated reference blend curve, a significant portion of LLDPE-2 crystallized at higher temperature (from region “B” to region “A” in Figure 11b). This was mainly due to the co-crystallization effect between HDPE-2 and LLDPE-2.

**Figure 12:**
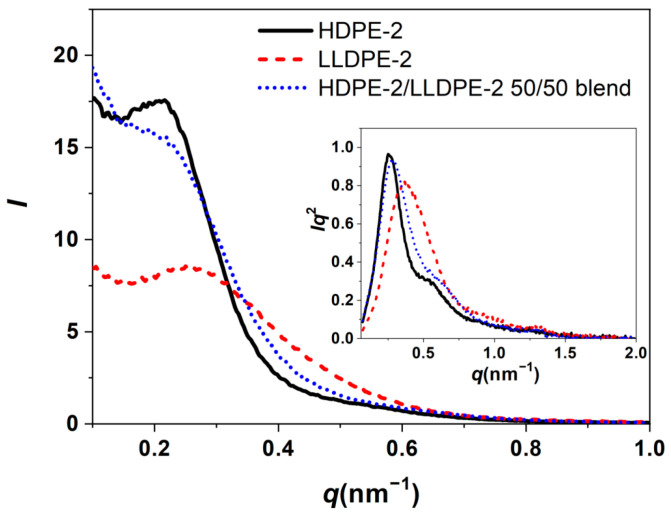
Linear and Lorentz-corrected scattering profiles of the neat HDPE-2, LLDPE-2, and the HDPE-2/LLDPE-2 50/50 blend.

**Figure 13:**
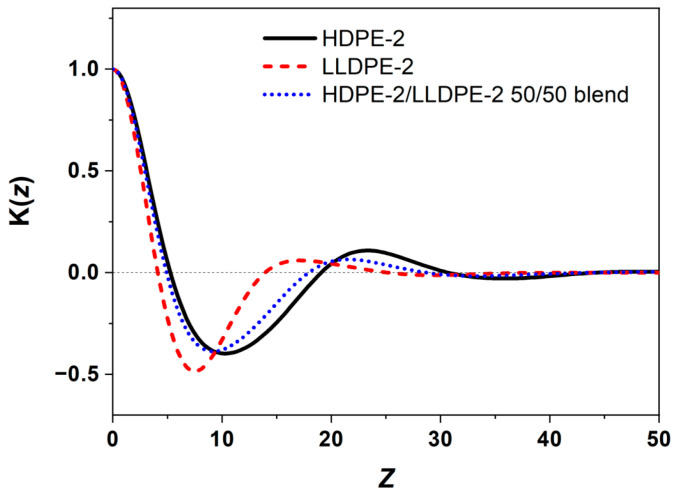
One-dimension correlation functions of the neat HDPE-2, LLDPE-2, and the HDPE-2/LLDPE-2 50/50 blend.

In study of the 50/50/HDPE-2/LLDPE-2 blend, it was observed that the crystalline peak around 65 °C during cooling was noticeably larger than that of the reference blend (region “D” in Figure 11b). This can be interpreted as follows: while most of the LLDPE-2 could co-crystallize with HDPE-2, the small fraction of highly branched LLDPE-2 with low crystalline ability could not participate in the co-crystallization process at higher temperatures with HDPE-2. During further cooling to low temperature range, there was much less LLDPE-2 crystallization going on for this small fraction of LLDPE-2 to participate. As a result, this fraction crystallized at a much lower temperature near 65 °C. In other words, co-crystallization with HDPE-2 caused fractionation of the crystallization of LLDPE-2. The crystallization structure of the HDPE-2/LLDPE-2 blend was studied by SAXS in comparison with the two neat components. The linear and Lorentz-corrected SAXS profiles of the blend were similar to those of HDPE-2, respectively, as shown in Figure 12, rather than an average of the two neat components. This suggested that the crystalline structure of the HDPE-2/LLDPE-2 blend was similar to that of HDPE-1. It can be understood from the crystallization study by DSC that HDPE-2 in the blend crystallized first in the blend, and most of the LLDPE-2 in the blend co-crystallized with HDPE-2 at a temperature near the crystallization temperature of the neat HDPE-2 (Figure 11a). In other words, the co-crystallization made the blend more like HDPE-2. The normalized one-dimension function of the HDPE-2/LLDPE-2 blend shown in Figure 13 was also similar to neat HDPE-2. The blend had a slightly smaller thickness of the crystalline phase (*L*_c_) and long spacing (*L*) than the neat HDPE-2, as shown in Table 9. This was consistent with the fact that the melting temperature of the blend (127.2 °C) was a little lower than that of the neat HDPE-2 (130.7 °C) owing to co-crystallization between HDPE-2 and LLDPE-2. However, the regularity of the lamella in the blend was not as good as in the neat HDPE-2, as evidenced by a less well-defined scattering peak in the linear SAXS profile, a broader scattering peak in the Lorentz-corrected SAXS profile, and a less well-defined peak in the one-dimensional correlation function. The results can be explained by the co-crystallization effect and a broader composition distribution of the blend than the neat HDPE-2, as shown in Figure 11a.

In summary, it can be seen from CRYSTAF in Figure 8a that the blend was a typical bimodal system with HDPE-2 and the fraction of high crystalline ability from the LLDPE-2 as one component, and a broad weaker crystalline ability of LLDPE-2 as another component. DSC and X-ray studies revealed that the HDPE-2 dominated the crystallization process and the structure formation with the participation of co-crystallization from the higher crystalline ability portion of the LLDPE-2. The weaker crystalline ability portion of the LLDPE-2 may need to crystallize within the main structure formed at a lower temperature.

Compared to the HDPE-2/LDPE blend, the difference in crystalline abilities between the components was much less in the HDPE-2/LLDPE-2 blend. The crystallization temperature of the neat LLDPE-2 was 107.2 °C, which was 8.3 °C lower than that of the neat HDPE-2. As a result, the HDPE-2/LLDPE-2 blend exhibited a more well-defined lamellar structure than the HDPE-2/LDPE blend. Considering both blends as a PE mixture, the composition of the HDPE-2/LDPE was much broader and bimodal (see Figure 5a), while the composition of the HDPE-2/LLDPE-2 blend was narrower (see Figure 11a). It can also be easily understood that the lamella that formed in the HDPE-2/LDPE blend was much less organized that that in the HDPE-2/LLDPE-2 blend.

As discussed in Section 3.1, Section 3.2 and Section 3.3 above, all three blends showed a certain degree of miscibility between the components. Interactions containing co-crystallization, nucleation, and fractionation occurred during crystallization processes, as evidenced by the DSC results. In the HDPE-2/LLDPE-2 50/50 blend, the difference in the crystallization temperature (*T*_c_) between the two components was the smallest among the three blends, at 8.3 °C. The miscibility between components in the HDPE-2/LLDPE-2 50/50 blend was the strongest, with co-crystallization mainly occurring in the blend and almost no separate stacks of LLDPE-2. In the HDPE-2/LDPE 50/50 blend, the difference in *T_c_* between the two components (22.2 °C) was much greater than the HDPE-2/LLDPE-2 50/50 blend, and some extent of co-crystallization still occurred in the blend. The crystallization of HDPE-2 also had a nucleation and fractionation effect on the crystallization of LDPE. The lamella packing of the HDPE-2/LDPE blend was more similar to that of neat HDPE-2 owing to HDPE-2’s stronger crystallizability compared to LDPE. In the LLDPE-2/LDPE blend, the difference in *T*_c_ (13.9 °C) was between that of the HDPE-2/LDPE 50/50 blend (22.2 °C) and the HDPE-2/LLDPE-2 blend (8.3 °C), as shown in Scheme 1. Co-crystallization, nucleation, and fractionation all occurred in the LLDPE-2/LDPE blend, as in the HDPE-2/LDPE blend. However, the crystallization structure of the LLDPE-2/LDPE blend was different from that of the HDPE-2/LDPE blend. In the HDPE-2/LDPE blend, the lamellar packing was more similar to that of the HDPE-2 component with its much stronger crystallizability. In the LLDPE-2/LDPE blend, however, the difference in crystallizability was not as large, and the lamellar packing was an average of the two neat components. Additionally, the extent of co-crystallization in the LLDPE-2/LDPE blend was less than that in the HDPE-2/LLDPE-2 blend.

## 4. Conclusions

A narrower distribution seen from CRYSTAF led to a more uniform thickness of crystal lamellae, resulting in a more ordered lamellar packing. It is understandable that a stronger crystalline ability represented the higher temperature peak from CRYSTAF corresponding to a higher melting point for the bulk crystallization from DSC, and a broader distribution in crystalline ability seen from CRYSTAF led to a broader melting peak for the bulk crystallization from DSC, such as in LLDPE-2 in Figure 1 and Figure 2. However, the crystalline ability distribution from CRYSTAF did not always have a good correlation with non-isothermal crystallization, which can be easily changed by the nucleation behavior of the PE resins.

The composition distribution of the neat PE and the binary blend were studied by CRYSTAF, and crystallization of the components in the dilute solution of blends for each molecule was almost isolated. Systematic and semi-quantitative analyses of the crystallization behaviors of three binary PE blends were carried out by DSC with hypothesized immiscible blends introduced for comparison. The PE molecules in the PE blends successively crystallized according to different crystalline abilities when the blends were cooled. Interactions including co-crystallization, nucleation, and fractionation between components in the blends were investigated by the difference in the crystallization behaviors of the blends compared with the reference immiscible blends, the extent of which was related to the difference in the crystallizability between components. The mutual influence during crystallization also indicated the miscibility between the components. The lamellar packing of the blends was related to the crystallization behaviors, and the crystalline structure strongly depended on the compositions. In the blend of HDPE with LLDPE or LDPE, the lamellar packing was more like that of the HDPE component with a much stronger crystallizability. In the LLDPE/LDPE blend, the lamellar packing was almost the average of the two neat components.

With the progress in catalyst technology and polymerization processes, the development of new high performance LLDPEs with multi-peak compositions is a trend. The results presented in this work aid in our understanding of the structure–property relationship of these resins. Furthermore, research on the interactions among components during crystallization and the final crystalline structures in these PE mixtures will remain an important and fundamental issue.

## Figures and Tables

**Table 1:** Material information of different polyethylene resins.

Type	Manufacture	Grade	Comonomer	Density ^(a)^ /(g/cm^3^)	${\bar{M}}_{w}$ **(×10^4^)**	*Đ*_M_^(b)^	Branching Content (/1000C)
HDPE-1	Sinopec	B5703	Propylene	0.960	19.9	10.71	0.4
HDPE-2	Sinopec	YEM-4902T	Butene	0.951	24.4	26.99	3
LLDPE-1	ExxonMobil	Enable 2010HA	Hexene	0.920	10.1	3.67	12.2
LLDPE-2	DOW	Dowlex 2045G	Octene	0.920	12.7	3.85	11.1
LDPE	Sinopec	LD607	——	0.921	8.6	5.23	13.6 ^(c)^

(a) 23 °C, data obtained from the producers; (b) the molecular weight dispersity ***Đ***_M_ *=* ${\bar{M}}_{w}/{\bar{M}}_{n}$; (c) short-chain branching: 11.2/1000C; long-chain branching with the carbon number greater than 6: 2.4/1000C).

**Table 2:** Characterization of the neat PEs during DSC cooling and subsequent heating at a given rate of 10 °C /min.

Sample	Higher *T*_c_/Lower *T*_c_ (°C)	*T*_m_ (°C)	Crystallinity (%)
HDPE-1	119.3/86.1	135.5	76.4
HDPE-2	115.5/79.8	130.7	69.0
LLDPE-1	102.4/66.4	115.3	41.8
LLDPE-2	107.2/61.7	123.9/122.4/109.4	40.9
LDPE	93.3/57.8	107.5	39.9

**Table 3:** Data of the long period (*L*), crystalline phase thickness (*L*_c_), and amorphous phase thickness (*L*_a_) obtained from the one-dimension correlation function.

Sample	*L* (nm)	*L*_c_ (nm)	*L*_a_ (nm)
HDPE-1	27.5	20.1	7.4
HDPE-2	23.2	16.5	6.7
LLDPE-1	14.6	5.3	9.3
LLDPE-2	17.1	5.7	11.4
LDPE	13.2	4.9	8.3

**Table 4:** DSC data of the HDPE-2/LDPE 50/50 blend compared with the neat components.

Sample	Higher *T*_c_/Lower *T*_c_ (°C)	*T*_m_ (°C)	Crystallinity (%)
HDPE-2	115.5/79.8	130.7	72
LDPE	93.3/57.8	107.5	42
HDPE-2/LDPE 50/50 blend	115.2/96.9/61.6	128.1/108.4	58

**Table 5:** Data of the long period (*L*), crystalline phase thickness (*L*_c_), and amorphous phase thickness (*L*_a_) obtained from the one-dimension correlation function.

Sample	*L* (nm)	*L*_c_ (nm)	*L*_a_ (nm)
HDPE-2	23.2	16.5	6.7
LDPE	13.2	4.9	8.3
HDPE-2/LDPE 50/50 blend	21.6	15.9	5.7

**Table 6:** DSC data of the LLDPE-2/LDPE 50/50 blend compared with the neat components.

Sample	Higher *T*_c_/Lower *T*_c_ (°C)	*T*_m_ (°C)	Crystallinity (%)
LLDPE-2	107.2/61.7	123.9/122.4/109.4	45
LDPE	93.3/57.8	107.5	42
LLDPE-2/LDPE 50/50 blend	106.9/95.2/59.5	123.1/120.9/106.4	44

**Table 7:** Data of the long spacing (*L*), crystalline phase thickness (*L*_c_), and amorphous phase thickness (*L*_a_) obtained from the one-dimension correlation function.

Sample	*L* (nm)	*L*_c_ (nm)	*L*_a_ (nm)
LLDPE-2	17.1	5.7	11.4
LDPE	13.2	4.9	8.3
LLDPE-2/LDPE 50/50 blend	15.1	5.3	9.8

**Table 8:** DSC data of the HDPE-2/LLDPE-2 50/50 blend compared with the neat components.

Sample	Higher *T*_c_/Lower *T*_c_ (°C)	*T*_m_ (°C)	Crystallinity (%)
HDPE-2	115.5/79.8	130.7	72
LLDPE-2	107.2/61.7	123.9/122.4/109.4	45
HDPE-2/LLDPE-2 50/50	114.1/67.0	127.2	56

**Table 9:** Data of the long period (*L*), crystalline phase thickness (*L*_c_), and amorphous phase thickness (*L*_a_) obtained from the one-dimension correlation function.

Sample	*L* (nm)	*L*_c_ (nm)	*L*_a_ (nm)
HDPE-2	23.2	16.5	6.7
LLDPE-2	17.1	5.7	11.4
HDPE-2/LLDPE-2 50/50	21.6	15.4	6.2

## Data Availability

The data presented in this study are available on request from the corresponding author.

## List of Abbreviations

PE	polyethylene
HDPE	high-density polyethylene
LLDPE	linear low-density polyethylene
LDPE	low-density polyethylene
CRYSTAF	crystallization analysis fractionation
TREF	temperature rising elution fractionation
DSC	differential scanning calorimetry
SAXS	small-angle X-ray scattering
SANS	small-angle neutron scattering
SALS	small-angle light scattering
TEM	transmission electron microscopy
